# Asymptomatic Dysphagia and Aspiration in Patients with Idiopathic Bronchiectasis

**DOI:** 10.1007/s00408-024-00683-5

**Published:** 2024-03-18

**Authors:** Tal Perluk, Eiman Abu Bandora, Ophir Freund, Tommy Jacob, Inbal Friedman Regev, Eyal Kleinhendler, Michal Shteinberg, Amir Bar-Shai, Yael Oestriecher-Kedem

**Affiliations:** 1 Institute of Pulmonary Medicine, Tel-Aviv Sourasky Medical Center, School of Medicine, Tel-Aviv University, 6 Weizman St., 6423906 Tel Aviv-Yafo, Israel; 2 Department of Otolaryngology, Head and Neck Surgery and Maxillofacial Surgery, School of Medicine, Tel-Aviv Sourasky Medical Center, Tel-Aviv University, Tel Aviv-Yafo, Israel; 3 Pulmonology Institute and CF Center, Carmel Medical Center, Haifa, Israel; 4 The B. Rappaport Faculty of Medicine, Technion-Israel Institute of Technology, Haifa, Israel

**Keywords:** Fiberoptic Endoscopic Evaluation of Swallowing, Bronchiectasis, Diagnosis, EAT-10 score, Aspiration

## Abstract

**Purpose:**

Although considered contributors to idiopathic bronchiectasis (IB), neither dysphagia nor silent aspiration have been systematically evaluated in IB patients. We aimed to explore the prevalence of asymptomatic dysphagia and silent aspiration in IB patients and to identify parameters predictive of their presence.

**Methods:**

This prospective cohort study included IB patients from our Pulmonary Institute without prior history of dysphagia and without prior dysphagia workup. Swallowing function was assessed by the Eating Assessment Tool (EAT-10) questionnaire and by the Fiberoptic Endoscopic Evaluation of Swallowing (FEES) test.

**Results:**

Forty-seven patients (31 females, mean age 67 ± 16 years) were recruited. An EAT-10 score ≥ 3 (risk for swallowing problems) was present in 21 patients (44.6%). Forty-two patients (89.3%) had at least one abnormal swallowing parameter in the FEES test. Six patients (12.7%) had a penetration aspiration score (PAS) in the FEES of at least 6, indicating aspiration. An EAT-10 score of 3 was found to be the ideal cutoff to predict aspiration in the FEES, with a good level of accuracy (area under the curve = 0.78, 95% CI 0.629–0.932, *p* = 0.03) and sensitivity of 83%. This cutoff also showed a trend towards a more severe disease using the FACED (forced expiratory volume, age, colonization with pseudomonas, extension of lung involvement, dyspnea) score (*p* = 0.05).

**Conclusion:**

Dysphagia is prevalent in IB and may be undiagnosed if not specifically sought. We recommend screening all patients with IB for dysphagia by the EAT-10 questionnaire and referring all those with a score of ≥ 3 to formal swallowing assessment.

## Introduction

Bronchiectasis is a chronic respiratory disease characterized by irreversible dilation of the bronchi, which can lead to chronic cough, sputum production and recurrent respiratory infections. Bronchiectasis commonly results from a variety of factors, such as chronic lung infections, cystic fibrosis, primary ciliary dyskinesia, inflammatory disorders and immune system abnormalities [1, 2]. Gatro-oesophageal reflux disease with aspiration of oesophageal content is a known comorbidity of bronchiectasis, however, current guidelines recommend investigating aspiration only in patients with symptomatic dysphagia or when there are other suggestive clinical features [3, 4]. Silent aspiration is defined as the inhalation of foreign material into the airways without overt signs or symptoms of aspiration [5]. Recurrent aspirations, as is the case with silent aspiration, is recognized to cause bronchiectasis, as also stated by the British thoracic society bronchiectasis in adults guidelines [4]. Oropharyngeal dysphagia (OD) is a common type of dysphagia referring to a disturbance in the oropharyngeal swallowing phase, which could also cause silent aspiration. Lung-related complications of aspiration include pneumonia and diffuse aspiration bronchiolitis, both could possibly lead to bronchiectasis [6]. Despite its potential impact on respiratory health, the prevalence of silent aspiration in patients with bronchiectasis is unknown and its clinical implications are not well understood and studied.

The term ‘‘idiopathic’’ is currently used to denote bronchiectasis of unknown origin (IB). The diagnosis of IB is based upon a combination of abnormal findings on clinical evaluation, imaging studies and laboratory tests, while ruling out known etiologies [3, 4]. In search for another potential etiology for bronchiectasis, we hypothesized that dysphagia and silent aspiration may be underlying factors and an undiagnosed cause of IB. Therefore, we aimed to explore the prevalence of silent aspiration in IB patients as a possible under-diagnosed cause of IB, and to identify parameters predictive for its occurrence. To the best of our knowledge, no earlier study had screened for asymptomatic dysphagia and silent aspiration in IB patients.

## Materials and Methods

### Study Design and Patients

This is a prospective cohort study conducted between May 2019 and July 2022 at the Tel-Aviv Sourasky Medical Center, a tertiary medical center in Tel-Aviv, Israel. Consecutive adult patients (age ≥ 18 years) with bronchiectasis were recruited during their visit to the Institute of Pulmonary Medicine. Only patients diagnosed with IB after a targeted work-up were recruited [7]. Considering the study aim, we decided to include only IB patients, which do not have other confounding etiologies to explain the presence of bronchiectasis. The workup included a chest computed tomography (CT) scan to confirm the diagnosis of bronchiectasis, a detailed medical history to exclude relevant and possible causes of bronchiectasis (such as rheumatoid arthritis, Sjögren’s syndrome, chronic obstructive pulmonary disease [COPD], allergic bronchopulmonary aspergillosis (ABPA), gastroesophageal reflux disease, stroke and inflammatory bowel disease), and laboratory tests (including a full blood cell count, serum total IgE, serum immunoglobulin G subclasses, immunoglobulin A, immunoglobulin M and sputum culture). Patients with prior stroke underwent an assessment by a senior neurologist and were included in the study only if there were no detectable neurologic deficits. Patients with COPD were excluded if the bronchiectasis diagnosis was in-adjacent or after the diagnosis of COPD. Patients who had a history of dysphagia or any reason for a prior dysphagia workup, were excluded. To verify these exclusion criteria, patients were interviewed prior to study inclusion, and their past medical records from prior hospitalizations and clinic visits were then reviewed. Patients with cognitive decline were excluded from study entry. Only the findings of patients who met the inclusion criteria, consented to participate in the study and completed the study protocol are included in this report.

The study was reviewed and approved by the Institutional Review Board, (TLV-0582-18). The study is reported in accordance with the Strengthening the Reporting of Observational Studies in Epidemiology (STROBE) guidelines.

### Study Protocol

After providing written informed consent, each participant filled in the Eating Assessment Tool (EAT-10) questionnaire, which is a 10-item self-assessment screening scale aimed at identifying individuals at high risk for swallowing disorders [8]. Each parameter is graded on a scale from 0 (no problem) to 4 (severe problem). The total EAT-10 score was the sum of the scores of all 10 parameters. A cutoff score of 3 was chosen in our study for the detection of dysphagia, in accordance with a meta-analysis that utilized the diagnostic accuracy of this cutoff [9]. Each participant’s bronchiectasis severity index, based upon the information derived from their medical interview and medical records, was determined on a scale of 0–7 (0–2 = mild; 3–4 = moderate: 5–7 = severe) using the FACED score [10]. The FACED score is based on five parameters to determine bronchiectasis severity: (1) forced expiratory volume in the first second (FEV1) value (≥ 50% = 0 points, < 50% = 2 points), (2) age (< 70 years = 0 points, ≥ 70 years = 2 points), (3) chronic lung colonization by Pseudomonas (1 point), (4) extent of lung involvement (1–2 lobes affected = 0 points, > 2 lobes affected = 1 point), and (5) dyspnea severity based on the modified Medical Research Council scale (mMRC) (0–2 = 0 points, 3–4 = 1 point). We extracted the spirometry results of included patients, with the nearest results to their inclusion chosen for analysis.

All included participants underwent a fiberoptic endoscopic evaluation of swallowing (FEES) test at the Voice and Swallowing Clinic. The evaluation was performed according to the accepted protocol by an experienced otolaryngologist and a speech pathologist specializing in dysphagia [11]. The FEES test was performed with the patient seated. A flexible fiberoptic laryngoscope connected to a camera and a digital recording video system was inserted through the nose to the hypopharynx to allow adequate visualization of the larynx and pharynx. The patients were instructed to swallow three food consistencies (pureed in the form of two spoons of apple sauce containing food coloring, solid in the form of two bites of a biscuit and liquid in the form of two spoons of food colored milk and, if there was no suspicion of aspiration, further two separate sips from a cup and one uninterrupted sip under visualization. The FEES test function parameters included: saliva stasis, abnormal sensation, spillage before swallowing, abnormal bolus location before swallowing, abnormal time to swallow reflex initiation, penetration (entrance of the bolus to the larynx), aspiration (entrance of the bolus to trachea), bolus residues, spillage after swallowing and ineffective cough reflux, hypopharyngeal reflux and oral motor function. We used the “touch method” to assess laryngeal sensation [12]. If no laryngeal adductor reflex (LAR), swallowing, or coughing was induced by touching the epiglottis or arytenoids with the tip of the fiberoptic laryngoscope, laryngeal sensation impairment was assumed.

We used two methods to quantify the FEES abnormalities. Each one of the 12 FEES test function parameters received a score of 0 (no abnormality) or 1 (abnormality). The sum of all abnormalities was calculated to yield a FEES test score between 0 and 12 for each patient. We also adopted the penetration aspiration score (PAS) [13] (used to grade dysphagia on videofluoroscopic swallow studies) to quantify the FEES test scores. The score is an 8-point ordinal scale, with 1 representing the least and 8 representing the highest or most severe score. A PAS score was calculated for each patient at the completion of each FEES test. The PAS score we chose for each patient for statistical analysis was the highest score of the entire FEES, independent of the viscosity of the food bolus or the phase of swallowing which it scored [14]. Due to the limitation of FEES, we did not score the “white-out” phase. PAS scores of 6 to 8 indicated the presence of aspiration (entrance of the bolus to the trachea), and PAS scores of 2 to 5 indicated penetration to the larynx.

### Statistical Analysis

Statistical analysis was performed using SPSS (IBM Corp. Released 2016. IBM SPSS Statistics for Windows, Version 24.0, Armonk, NY). All statistical tests were two-tailed, and *p* < 0.05 was considered significant. Categorical variables were described as frequency and percentage. Continuous variables were evaluated for normal distribution and described as median and interquartile range or mean and standard deviation (SD). Categorical variables were compared with the chi-square test and continuous variables were compared with the Mann–Whitney test. Correlations between non-parametric variables were assessed by means of Spearman’s rank test.

A receiver operating characteristic curve was generated for the estimation of The EAT-10 score’s predictive ability for aspiration (a PAS score of ≥ 6). This allowed for the calculation of area under the curve (AUC), and for determining the optimal cutoff point for the EAT-10 score. Sensitivity, specificity, positive predictive value, negative predictive value as well as confidence interval (CI) were calculated.

## Results

Forty-seven patients (31 females, mean age 67 ± 16 years) met the inclusion criteria during the study period, consented to participate in the study and completed the study protocol. Their demographic and clinical characteristics are summarized in Table 1. Three patients with a cerebral vascular accident (CVA) history were included in the study after their neurologist confirmed that there were no apparent sequelae of their CVA. Five subjects (11%) had history of asthma, which was mild-moderate and well-controlled in all cases. Bronchiectasis distribution was in the upper or middle lobes in 57% of the cases, lower lobes in 34%, and in both upper and lower lobes in 9%. The mean forced expiratory volume (FEV1) was 81.4 ± 14%predicted, mean forced vital capacity (FVC) was 89.1 ± 15%predicted, and the mean FEV1/FVC was 0.75 ± 0.09. The median (IQR) FACED score was 2 (0–3). Based in the FACED score, thirty-three patients (70.2%) had mild, 12 (25.5%) had moderate and 2 (4.2%) had severe disease.

**Table 1:** Cohort characteristics

Variable	Study cohort *N* = 47 (%)
Age	67 + 16
Female sex	31 (66)
Gastroesophageal reflux	17 (46)
Prior/current smoking	16 (34)
Concurrent lung disease	
Asthma	5 (11)
COPD^a^	2 (4)
Interstitial lung disease	1 (2)
Prior ischemic stroke	3 (6)
FEV1%predicted	81.4 ± 14
FVC %predicted	89.1 ± 15
FEV1/FVC	0.75 ± 0.09
*Bronchiectasis characteristics*	
Lung zone	
Lower lobes	16 (34)
Upper and middle lobes	27 (57)
Both zones	4 (9)
FACED score	
Mild (0–2)	33 (70)
Moderate (3–4)	12 (26)
Severe (≥ 5)	2 (4)
Chronic bacterial colonization	18 (39)

^a^In both cases the diagnosis of bronchiectasis was made more than 5 years before the diagnosis of COPD

### EAT-10 Scores

The median (IQR) EAT-10 score was 2 (0–8). Twenty-one patients (44.6%) had an EAT-10 score ≥ 3. A weak but statistically significant correlation was found between a higher EAT-10 score and a higher FACED score (*p* = 0.028, *r* = 0.32). In addition, patients with an EAT-10 score of above 3 show a trend towards higher FACED scores (*p* = 0.05).

### FEES Test Results

The FEES test results are summarized in Tables 2 and 3. At least one swallowing abnormality was found in 42 patients (89.3%). Nineteen patients (40.4%) exhibited penetration-related abnormalities and 6 patients (12.7%) exhibited aspiration-related abnormalities. No patient displayed either an ineffective cough or abnormal bolus location before swallowing. Nineteen of the 42 patients with at least one swallowing abnormality had an EAT-10 score > 3. No correlations were found between the FEES test score of individual patients and their FACED or EAT-10 scores (*p* = 0.615 and *p* = 0.386, respectively) or any of the baseline or clinical characteristics.

**Table 2:** Fiberoptic endoscopic evaluation of swallowing test results

Parameter	Total, *N* = 47 (%)
Saliva stasis prior to examination	7 (15)
Abnormal sensation	7 (15)
Spillage before swallowing	3 (6)
Abnormal bolus location before swallowing	0 (0)
Abnormal time to swallow reflux initiation	1 (2)
Penetration	19 (40)
Aspiration	6 (13)
Bolus residue	22 (47)
Spillage after swallowing	12 (25.5)
Ineffective cough	0 (0)
Hypopharyngeal reflux	6 (13)
Abnormal oromotor function	10 (21)

**Table 3:** Distribution of fiberoptic endoscopic evaluation of swallowing (FEES) test abnormalities

FEES abnormalities	Total, *N* = 47 (%)
0	5 (10.6)
1	18 (38.3)
2	11 (23.4)
3	4 (8.5)
4	5 (10.5)
5	2 (4.2)
6	2 (4.2)

The PAS scores are presented in Table 4. Six patients (12.7%) had a PAS score of ≥ 6, signifying aspiration. A comparison of the patients’ PAS and FACED scores revealed no correlation between them (*p* = 0.329).

**Table 4:** Penetration aspiration score

Queried items	Total, *N* = 47 (%)
1-Material does not enter airway	26 (55)
2-Material enters the airway, remains above the vocal folds and is ejected from the airway	13 (28)
3-Material enters the airway, remains above the vocal folds and is not ejected from the airway	0 (0)
4-Material enters the airway, contacts the vocal folds and is ejected from the airway	1 (2)
5-Material enters the airway, contacts the vocal folds and is not ejected from the airway	1 (2)
6-Material enters the airway, passes below the vocal folds and is ejected into the larynx or out of the airway	6 (13)
7-Material enters the airway, passes below the vocal folds and is not ejected from the trachea despite effort to eject	0 (0)
8-Material enters the airway, passes below the vocal folds and no effort is made to eject	0 (0)

### Correlation Between EAT-10 and PAS Scores

**Fig. 1:**
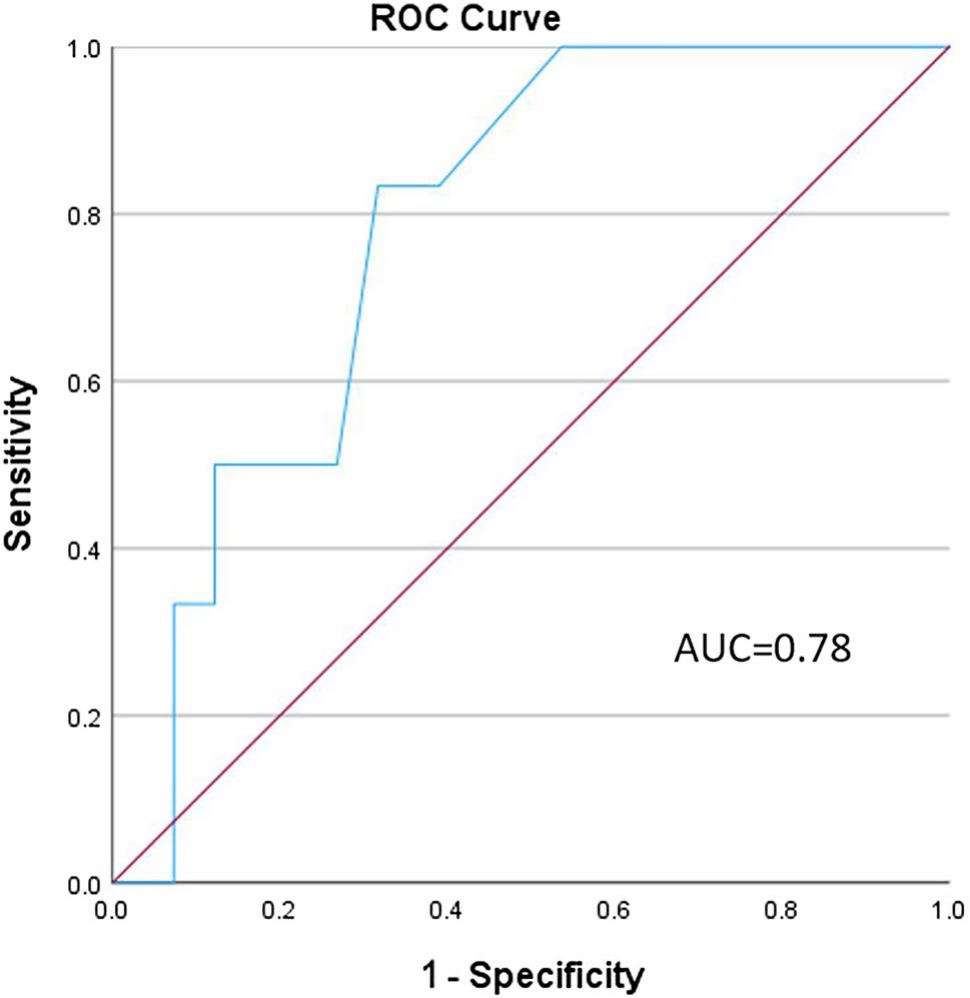
Receiver operating characteristic (ROC) curve for the utility of EAT-10 in detecting aspiration by FEES in patients with idiopathic bronchiectasis

The diagnostic accuracy of EAT-10 in detecting aspiration was calculated, and it is represented by the receiver operating characteristic curve in Fig. 1. We found that the EAT-10 score had a good predictive ability for a PAS score of above 6, indicating aspiration (AUC = 0.78, SD = 0.077, confidence interval 0.629–0.932, *p* = 0.028). The optimal cutoff value for the EAT-10 score to predict aspiration was 3, with sensitivity, specificity, positive predictive value and negative predictive value of 83, 60, 23, and 96%, respectively.

## Discussion

Bronchiectasis is a chronic and debilitating respiratory condition with considerable morbidity and impaired health-related quality of life [15, 16]. In this prospective study we aimed to search another potential cause of IB, and found that asymptomatic dysphagia is prevalent in idiopathic bronchiectasis, thus screening for its existence is recommended. Although aspiration is a suspected cause of bronchiectasis, the rate of silent aspiration and asymptomatic dysphagia in IB patients is unknown, and to the best of our knowledge, studies of screening for such pathology are scarce. Over the past two decades, the study of oropharyngeal dysphagia has been approached from various disciplines and found to correlate with an increased risk of complications, including aspiration pneumonia, malnutrition, dehydration, and death [5, 17]. An association between OD and chronic pulmonary disorders was found, mainly COPD and dysphagia, and showed to contribute to COPD morbidity [18–20]. To the best of our knowledge there has been no previous study on the association between dysphagia and IB, and we believe that our study is unique by investigating the prevalence of dysphagia and silent aspiration specifically in an IB patient population.

Our study’s main finding is the high prevalence of dysphagia in our IB patient population. Forty-two of our patients (89.3%) exhibited dysphagia during FEES testing. Also, using FEES we could not assess aspiration or penetration during the “white-out” phase of swallowing, possibly leading to their under-estimation. In contrast, the reported prevalence of dysphagia ranges between 2 and 20% [21]. We speculate that such a high prevalence of dysphagia in our cohort could suggest a contributing role of dysphagia to the pathogenesis of IB. Of note, 19 of the 42 patients (44%) with at least one abnormal FEES test parameter had an EAT-10 score < 3 (44%), illustrating the high incidence of asymptomatic dysphagia in IB patients. This finding is in line with the observation that dysphagia often remains under-reported and underestimated in patients with bronchiectasis [22, 23].

Aspiration is a preventable cause for bronchiectasis, with dysphagia being a major etiology for aspiration [17]. Although evidence on this topic is scarce, aspiration is thought to cause bronchiectasis by several mechanisms. First, inhalation of oropharyngeal secretions colonized by pathogenic bacteria could lead to an acute infectious process, known as aspiration pneumonia [24], which by itself could lead to bronchiectasis (i.e. post-infectious bronchiectasis) [25]. By occurring repeatedly, this could also result in chronic airway infection and inflammation, and ultimately in bronchiectasis [26]. Second, inhaling gastric contents such as gastric acid and digestive enzymes (as in cases of gastroesophageal reflux disease) may result in chemical pneumonitis, with complications including airway obstruction and severe inflammatory response [27, 28]. Third, diffuse aspiration bronchiolitis, although a relatively rare sequela, often includes bronchiectasis [6]. Finally, malnutrition itself, which is prevalent in cases of severe dysphagia and aspirations, is thought to contribute to bronchiectasis by impaired immune function [25, 29]. Of note, we did not find prior evidence on the relevance of the specific aspirated content or it size in relation to bronchiectasis occurrence.

Our study’s results are in agreement with those of other studies in suggesting that the EAT-10 questionnaire can be utilized to detect patients with a high probability of sustaining an aspiration event [30]. Regan et al. reported a sensitivity of 91.7 and a specificity of 77.8 with a cut-off of > 9 in EAT-10 for detecting aspirating COPD patients. Cheney et al., used a cut-off of 16 with 71% sensitivity and 53% specificity to detect patients with aspiration in a general dysphagia patient group [31]. Belafsky et al., suggested that an EAT-10 score of 3 or above is abnormal [8]. This cut-off of 3 is consistent with the results of our study. Our study suggests that EAT-10 can be used to predict aspiration in IB patients as indicated by the high AUC of 0.78. Although the negative predictive (NPV) value of 96% with a cut-off of 3 translates into a low false-negative rate, suggesting that EAT-10 may be used as a screening tool for dysphagia in IB patients, the high NPV could be an overestimation due to our relatively small patient cohort.

Swallowing evaluation by instruments in the form of FEES and videofluoroscopic swallow studies are essential to assess swallowing efficacy and the status of airway protection. Although these methods are the gold standards for swallowing evaluation, there might be some limitations for their incorporation in the daily practice of busy clinics since they require special equipment, facility, trained staff and time [32, 33]. As such, they would not be suitable as screening tools of aspiration risk. In contrast, the high sensitivity of the EAT-10 questionnaire in our study suggests that it may be used as a screening tool to predict dysphagia and aspiration in patients with IB, and that those with an EAT-10 score < 3 can be excluded from further dysphagia workup. Interestingly, we observed a positive correlation between bronchiectasis severity and the EAT-10 score, suggesting that dysphagia could not only promote bronchiectasis but also contribute to severity. However, the finding was not reproduced by abnormalities in FEES. This could also be attributed to the relatively small patient cohort.

Several methodological limitations to our study bear mentioning. Although we included consecutive consenting patients, the relatively small cohort size and single-center setting limits the generalizability of our findings. Another limitation is a lack of a healthy control group as well as other aetiologies of bronchiectasis. While these concerns are reasonable when considering the EAT-10 as a stand-alone test, they do not diminish its value as a screening instrument to identify individuals at risk of aspiration who necessitate a more comprehensive investigation.

In conclusion, dysphagia is prevalent in IB patients and may be associated with bronchiectasis severity. Silent dysphagia and aspiration may go undiagnosed if not specifically sought during their clinical examination. We hypothesize that silent aspiration may represent an underdiagnosed cause of bronchiectasis, and found its high prevalence in our cohort. Identification of swallowing abnormalities in patients with IB and referral for appropriate dysphagia management can help guide the implementation of appropriate therapeutic interventions to prevent future serious dysphagia complications and further deterioration in the patient’s health. The inclusion of an easily applicable short and reliable self-reporting questionnaire could aid in detecting such abnormalities. We recommend the use of the EAT-10 questionnaire for screening all IB patients for dysphagia and referring those whose score is ≥ 3 to formal swallowing assessment. Future studies are warranted to examine the role of measures to prevent the occurrence of dysphagia among IB patients as manifested by disease severity, morbidity, and mortality and healthcare costs**.**

## Data Availability

The authors confirm that the data supporting the findings of this study are available within the article [and/or] its supplementary materials.
